# N^ε^-lysine acetylation of the histone-like protein HBsu influences antibiotic survival and persistence in *Bacillus subtilis*

**DOI:** 10.3389/fmicb.2024.1356733

**Published:** 2024-05-21

**Authors:** Rachel A. Carr, Trichina Tucker, Precious M. Newman, Lama Jadalla, Kamayel Jaludi, Briana E. Reid, Damian N. Alpheaus, Anish Korrapati, April E. Pivonka, Valerie J. Carabetta

**Affiliations:** ^1^Department of Biomedical Sciences, Cooper Medical School of Rowan University, Camden, NJ, United States; ^2^Rowan-Virtua School of Osteopathic Medicine, Stratford, NJ, United States

**Keywords:** acetyl, acetylation, antibiotic resistance, bacteria, nucleoid-associated protein, tolerance, persisters

## Abstract

N^ε^-lysine acetylation is recognized as a prevalent post-translational modification (PTM) that regulates proteins across all three domains of life. In *Bacillus subtilis*, the histone-like protein HBsu is acetylated at seven sites, which regulates DNA compaction and the process of sporulation. In Mycobacteria, DNA compaction is a survival strategy in response antibiotic exposure. Acetylation of the HBsu ortholog HupB decondenses the chromosome to escape this drug-induced, non-growing state, and in addition, regulates the formation of drug-tolerant subpopulations by altering gene expression. We hypothesized that the acetylation of HBsu plays similar regulatory roles. First, we measured nucleoid area by fluorescence microscopy and in agreement, we found that wild-type cells compacted their nucleoids upon kanamycin exposure, but not exposure to tetracycline. We analyzed a collection of HBsu mutants that contain lysine substitutions that mimic the acetylated (glutamine) or unacetylated (arginine) forms of the protein. Our findings indicate that some level of acetylation is required at K3 for a proper response and K75 must be deacetylated. Next, we performed time-kill assays of wild-type and mutant strains in the presence of different antibiotics and found that interfering with HBsu acetylation led to faster killing rates. Finally, we examined the persistent subpopulation and found that altering the acetylation status of HBsu led to an increase in persister cell formation. In addition, we found that most of the deacetylation-mimic mutants, which have compacted nucleoids, were delayed in resuming growth following removal of the antibiotic, suggesting that acetylation is required to escape the persistent state. Together, this data adds an additional regulatory role for HBsu acetylation and further supports the existence of a histone-like code in bacteria.

## 1 Introduction

Antibiotic resistance is a pressing concern in global public health, created by the rapid acquisition of resistance genes by bacteria. According to the 2019 Center for Disease Control and Prevention (CDC) antibiotic resistance threats report, more than 2.8 million antibiotic-resistant infections occur each year, leading to more than 35,000 deaths (CDC, 2019). There is a direct relationship between the use of antibiotics and the emergence of drug-resistant bacterial strains (Llor and Bjerrum, 2014). Drug resistance may evolve by *de novo* mutations or be acquired by horizontal gene transfer (HGT), which occurs via conjugation, bacteriophage transduction, or transformation, all resulting in the recombination of foreign DNA into host chromosomes (Sun, 2018; Lerminiaux and Cameron, 2019). HGT is a significant clinical concern because the transfer of resistance genes can occur between environmental or human-associated bacteria and pathogens, which then promotes the dissemination of resistance. These resistance genes are selected for when a population of bacteria is challenged by antibiotic exposure, which allows such resistant strains to dominate the population.

From a genetically identical population of cells, different subpopulations can have different growth rates and gene expression patterns, and the selection of these subpopulations contributes to antibiotic survival. A common physiologic characteristic of bacteria is dormancy, which is a state they enter when encountering external biological stressors. This feature allows specialized cells to survive in a hostile environment, where they enter a cycle of reduced metabolic activity and activate protective stress responses, such as the general stress or SOS response (Rittershaus et al., 2013; Harms et al., 2016). The presence of true resistance genes is irrelevant in such populations. This phenomenon is referred to as bacterial persistence. In a population, persister cells are spontaneously formed at a basal rate and this rate is influenced by adverse environmental conditions, such as a hostile host environment or sublethal concentrations of antibiotics. Persister formation occurs devoid of any new genetic modifications. Instead, a shift in global gene expression is responsible, creating a dormancy-related profile characterized by the inhibition of genes responsible for cell growth and division, while increasing the expression of stress-related genes (Harms et al., 2016). Even small variations in gene expression can cause the rise of antibiotic-tolerant subpopulations. Following removal of the stress, persisters escape from the dormant state and return to their normal metabolic functions. This capability of persisters to alternate between an active and inactive state is a key factor of antibiotic ineffectiveness and leads to prolonged and relapsing infections (Gollan et al., 2019).

N^ε^-lysine acetylation is recognized as a prevalent post-translational modification (PTM) that regulates proteins across all three domains of life. The level of acetylation is controlled by the action of lysine acetyltransferases (KATs), which transfer an acetyl group from a donor molecule, usually acetyl-CoA, to the target lysine sidechain amine. This action is reversed by the deacetylases, which are either Zn^+^-dependent lysine deacetylases (KDACs) or NAD^+^-dependent sirtuins (Christensen et al., 2019b). Some known functions of acetylation in bacteria are to control enzymatic activity, modulate protein stability, and regulate gene expression (Carabetta and Cristea, 2017; VanDrisse and Escalante-Semerena, 2019; Christensen et al., 2019a,b). In eukaryotes, the histones are responsible for chromatin compaction and regulation of various DNA transactions, such as DNA replication and gene expression. The N-terminal tails of the histones are highly modified by PTMs, which correlates to the degree of gene expression (Bannister and Kouzarides, 2011). This regulatory process has been referred to as the “histone code.” In general, lysine acetylation is associated with chromatin loosening and allows the DNA to be more accessible for RNA polymerase, which promotes transcription. The removal of the acetyl groups restores the positively charged lysine residues and strengthens the interaction between histones and DNA, which reduces gene expression (Strahl and Allis, 2000; Patel and Wang, 2013). Therefore, the level of chromatin compaction is correlated with the level of gene expression.

Bacteria have nucleoid-associated proteins (NAPs) that are considered functional equivalents of histones and are responsible for the compaction of the chromosome and regulation of DNA processes (Ohniwa et al., 2011). The HU family, also known as the DNABII family, are the most widely conserved NAPs in bacteria. The HU family of proteins constitutes of a class of non-specific, DNA-binding proteins that actively participate in a wide range of biological processes, such as protecting the chromosome from thermal denaturation, DNA compaction, and gene expression (Stojkova et al., 2019; Carabetta, 2021). In *Bacillus subtilis,* the HU-family ortholog is HBsu, which is acetylated at seven lysine residues *in vivo* (Carabetta et al., 2019). Many of the modification sites of these proteins are predicted to have direct contact with DNA, likely influencing their DNA binding activity. Acetylation of HBsu regulates DNA compaction and the process of sporulation (Carabetta et al., 2019; Luu et al., 2022). As HBsu is the major NAP in *B. subtilis* (Micka and Marahiel, 1992; Ohniwa et al., 2011), additional processes which involve chromosomal dynamics or gene expression are likely also influenced by its acetylation.

In Mycobacteria, the HU-family ortholog is HupB, which is non-essential in *Mycobacterium smegmatis* (Sakatos et al., 2018). In *M. smegmatis*, the chromosomal DNA is diffusely spread throughout the cell, and when these bacteria are exposed to the antibiotic fusidic acid, their chromosome condenses into a single, dense structure (Scutigliani et al., 2018). This behavior was also observed in *Mycobacterium tuberculosis* cells that were treated with a variety of antibiotics. DNA condensation decreases over time during drug exposure, indicating that drug-induced DNA condensation is reversible. Therefore, DNA condensation likely represents an important survival strategy in the presence of environmental stresses in Mycobacteria. In further support of this, DNA condensation was also observed in response to nutrient starvation (Scutigliani et al., 2018). As NAPs are responsible for chromosomal compaction in bacteria, they are likely important regulatory factors of this stress response. Sakatos et al., using high-throughput microfluidic imaging, observed multiple drug-resistant subpopulations when *M. smegmatis* cells were exposed to sub-lethal levels of isoniazid. These subpopulations, referred to as large and small colony variants (LCVs and SCVs, respectively), differ in cell size, division times, and transcriptional profiles. The differences between these two populations are not due to gene mutations, but rather epigenetics. It was observed that following isoniazid exposure, a *hupB* deletion strain resulted in a specific loss of drug-resistant subpopulations and did not influence the growth kinetics of the entire population. RNA-seq analysis revealed that the loss of *hupB* influences gene expression, with the upregulation of 77 genes (Sakatos et al., 2018). In *Mycobacterium* spp., it was found that when HupB is acetylated DNA binding affinity is decreased (Ghosh et al., 2016) and deacetylation has the opposite effect (Anand et al., 2017). HupB contains six methylated and acetylated sites, and three were identified that impacted DNA binding (Sakatos et al., 2018). Mutation of one of the identified lysine sites to the unmodified mimic arginine (K86R), resulted in a significant reduction of SCVs when exposed to isoniazid in comparison to wild-type. These findings suggest that epigenetic regulation in bacteria, involving the modifications of histone-like proteins, have global effects on gene expression that can influence antibiotic resistance and other essential cellular properties.

We hypothesized that acetylation of the histone-like proteins in Gram-positive bacteria play similar roles as they do in Mycobacteria. Thus, we determined the role that HBsu acetylation plays in *B. subtilis* during antibiotic challenge. We found that like *M. smegmatis* and *M. tuberculosis*, wild-type cells compact their nucleoids upon challenge with the aminoglycoside antibiotic kanamycin and that deacetylation of HBsu at specific sites is required for this compaction. We propose that nucleoid compaction is a survival strategy of bacteria when challenged with specific antibiotics and that HU family proteins may be important mediators of this response. We next examined the role of HBsu acetylation during drug survival by performing time-kill assays. We utilized a collection of HBsu mutants that contain lysine substitutions that mimic the acetylated (glutamine) or unacetylated (arginine) forms of the protein. Interfering with HBsu acetylation led to faster killing kinetics than wild-type. However, the number of cells that survived following 2 h of kanamycin or vancomycin exposure were similar among all strains, possibly representing persisters. Finally, we determined that interfering with the acetylation status of HBsu led to an increase in persister cell formation, following 5 h of exposure to kanamycin. This was different than what was observed for *M. smegmatis*. Although there was an increased number of persisters, the recovery from the semi-dormant persistent state was significantly delayed for many acetylation mutant strains. Together, this data adds an additional regulatory role for HBsu acetylation in survival following drug exposure, which further supports the existence of a histone-like code in bacteria.

## 2 Materials and methods

### 2.1 Bacterial strains, media, and growth conditions

All strains used in this study were constructed as previously described (Carabetta et al., 2019) and are listed in Table 1. These mutant strains have an identified lysine acetylation site (K3, K18, K37, K41, K75, K80, and K86) mutated to either glutamine (acetylated mimic) or arginine (deacetylated mimic) at the native locus. Liquid and agar Luria Broth (LB) were prepared according to standard protocols. Bacteria were grown in LB media at 37°C with aeration, with growth monitored by a Klett colorimeter. When appropriate, kanamycin, vancomycin, and tetracycline were added at final concentrations of 5, 12.5, and 25 μg/mL, respectively.

**Table 1:** Strains used in this study.

Strain	Relevant genotype^1^	Source/Ref
BD630	*his leu8 metB5*	Lab strain
BD7484	*hbsK80R*	Carabetta et al. (2019)
BD7493	*hbsK86Q*	Carabetta et al. (2019)
BD7506	*hbsK86R*	Carabetta et al. (2019)
BD8119	*hbsK37Q*	Carabetta et al. (2019)
BD8120	*hbsK37R*	Carabetta et al. (2019)
BD8147	*hbsK41Q*	Carabetta et al. (2019)
BD8148	*hbsK41R*	Carabetta et al. (2019)
BD8190	*hbsK18R*	Carabetta et al. (2019)
BD8219	*hbsK18Q*	Carabetta et al. (2019)
BD8333	*hbsK75R*	Carabetta et al. (2019)
BD8387	*hbsK3R*	Carabetta et al. (2019)
BD8398	*hbsK75Q*	Carabetta et al. (2019)
BD8576	*hbsK80Q*	Carabetta et al. (2019)
BD8577	*hbsK3Q*	Carabetta et al. (2019)

^1^All strains are in the BD630 background.

### 2.2 Fluorescence microscopy and data analysis

Cells were grown overnight at 30°C on LB plates and inoculated into fresh media the next morning. Specifically, cells were pre-grown in 5 mL LB for 2 h and after this period, the culture was split in half and subcultured into LB with or without kanamycin, tetracycline, or vancomycin. This extended pre-growth period allows for cells to enter exponential growth phase and increases cell numbers for subsequent analysis. After 20 min of growth, 300 μL of cells were harvested and nucleoids stained with a final concentration of 2 mM 4′, 6-diamidino-2-phenylindole (DAPI) for 5 min at room temperature. Cells were collected by centrifugation, washed once 300 μL of phosphate buffered saline (PBS, 10 mM potassium phosphate, pH 7.4, 0.15 M NaCl), and resuspended in 100 μL of PBS. 1 μL of cells was placed on 1% agarose pads for fluorescence microscopy, performed using a Nikon Eclipse Ti2 inverted wide-field microscope. Measurement of nucleoid area was performed using the NIS-Elements AR (Nikon) automated General Analysis feature (version 5.21.02). Using this software, thresholds based on DAPI intensity were manually applied to create a binary layer that identified the nucleoids as regions of interest for automated analysis. After initial thresholding was applied for wild-type cells that clearly separated the nucleoids from the background, the same filters were used for all images. The general analysis tool was used to measure nucleoid length, width, and area. At least 800 cells were analyzed for each strain, including multiple images from at least four biological replicates. The measurements generated were exported to Microsoft Excel for further processing. Large nucleoid lengths (> 3.2 μM) were excluded from analysis, which likely represented out of focus cells or those not differentiated as individual cells by the automated analysis program. The cutoffs for length and width measurements were determined as described previously (Carabetta et al., 2019). For each strain and growth condition, cumulative distribution plots were made by sequentially organizing the calculated nucleoid areas from low to high and plotting those values against their cumulative fraction. The cumulative distribution curves were plotted using the ggplot2 package in R (version 4.3.2, R Core Team, 2021). Nucleoid area distributions were compared between mutants and the wild-type in the presence or absence of kanamycin. The nonparametric, Kolmogorov–Smirnov test was used to determine if the two comparative samples came from the same distribution (Karson, 1968) and was performed in R, using the stats package. A *p* value <0.05 was considered statistically significant.

### 2.3 Time-kill assays

Cells were grown overnight at 30°C on LB plates, inoculated into LB media the next morning, and pre-grown for 2 h at 37°C, with aeration, as above. Following pre-growth, cells were serially diluted and plated on LB plates for initial cell count determination. Next, cells were diluted into flasks containing LB with or without kanamycin, tetracycline, or vancomycin. Growth was monitored by Klett colorimetry. Every 30 min for 2 h, cells were serially diluted and plated on LB plates to determine cell survival following antibiotic exposure. Percent survival was determined as colony-forming units (CFUs)/mL following antibiotic treatment/initial colony counts (CFUs/mL). All experiments were completed at least three independent times.

### 2.4 Persistence assays

Cells were grown overnight at 30°C on LB plates, inoculated into LB media the next morning, and pre-grown for 1 h at 37°C, with aeration to allow for entry into exponential growth phase. Following pre-growth, kanamycin was added to each culture. After 5 h of growth in the presence of antibiotic, cells were serially diluted and plated on LB plates for determination of colony counts (CFU/mL) to identify the surviving persistent population (Windels et al., 2019). All experiments were completed at least three independent times.

### 2.5 Persistence recovery assays

Cells were pre-grown in 1 mL of LB media for 1 h. Following pre-growth, kanamycin or vancomycin was added and cultures incubated overnight at 37°C, with aeration, to ensure that only viable, persistent cells would be assayed. The next morning, cells were washed three times with 1 mL LB media lacking antibiotics, resuspended in 1 mL LB, and 150 μL were added per well in a white, 96-well optical plate (ThermoFisher Scientific), in technical triplicates. The plate was sealed with a Breath-Easy sealing membrane [Research Products International (RPI)] and grown with shaking at 37°C in a Varioskan Lux (ThermoFisher Scientific) plate reader. Optical density measurements at 600 nm (OD_600_) were recorded every 10 min for 8 h. A background subtract step, using the data from wells containing only sterile media, was performed using SkanIT software (version 7.0.2), before exportation to Microsoft Excel for further analysis. Each experiment was completed two independent times.

## 3 Results

### 3.1 Wild-type cells compact their nucleoids upon exposure to kanamycin

**Figure 1:**
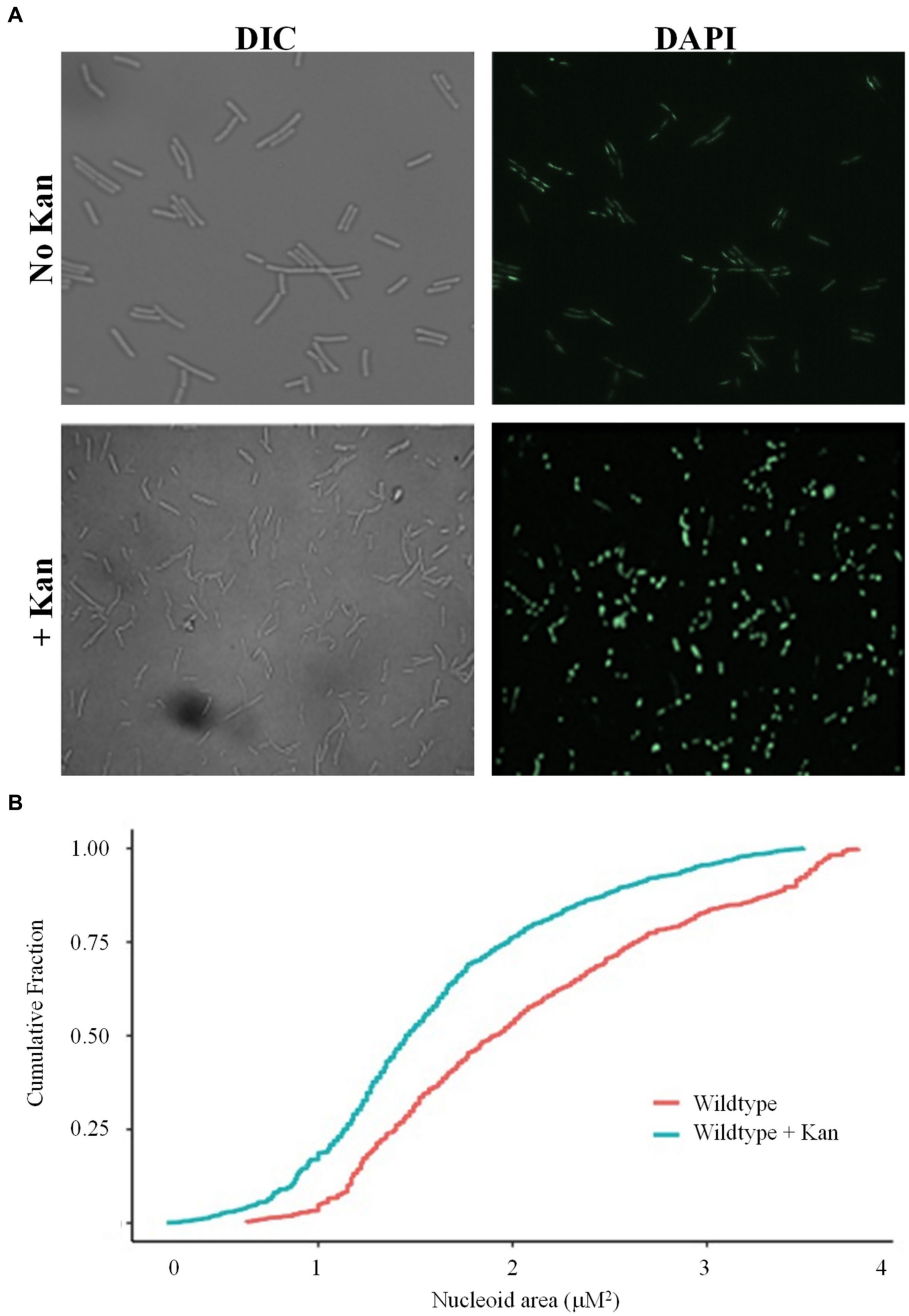
Wild-type cells compact their nucleoids in response to kanamycin challenge. **(A)** Wild-type cells (BD630) were pre-grown in LB media for 2 h, then incubated with or without 5 μg/mL kanamycin for 20 min, and nucleoids stained with DAPI. Representative microscopy images are displayed. DIC, Differential interference contrast. **(B)** Cumulative distribution plots are displayed, where the 50th percentile represents the median of the population distribution. Nucleoid areas of at least 800 cells were analyzed. The distributions with and without kanamycin were significantly different (*p* value = 2.54e^−7^), as determined by the Kolmogorov–Smirnov test.

It was previously demonstrated that when challenged with antibiotics, Mycobacteria respond by compacting their DNA, possibly for additional protection from drug-induced oxidative stress (Scutigliani et al., 2018). However, it is unknown if this is a universal bacterial response to drug challenge. Wild-type *B. subtilis* cells were harvested following 20 min of growth in the presence or absence of the aminoglycoside antibiotic kanamycin, and nucleoids visualized by staining with DAPI. In LB media, before cell division, the nucleoids were diffused and appeared to be almost the same size as the cell (Figure 1A). However, when wild-type cells were challenged with kanamycin, the nucleoids became more rounded and compacted, and stained more intensely with DAPI (Figure 1A), in agreement with our previous observations (Carabetta et al., 2019). Quantification of the nucleoid areas of cells in the presence of kanamycin showed that the population distribution curve was shifted to the left when compared to cells in the absence of the drug, indicating a significant reduction in nucleoid size across the population (Figure 1B, *p* value = 2.54e^−7^). Next, we looked at the response of wild-type cells to another bactericidal antibiotic, the glycopeptide vancomycin, and a bacteriostatic drug tetracycline. In response to tetracycline, the distribution of nucleoid areas was similar to wild-type without drug (Supplementary Figure S1, *p* value = 0.6385), and no compaction was observed. For vancomycin, the distribution was statistically different from wild-type without drug (*p* value = 0.0004), but was slightly shifted to the right, rather than compacted (Supplementary Figure S1). We speculate that these differences might be related to the mechanism of killing, specifically the generation of reactive oxygen species (ROS), which will be discussed below. This data indicated that *B. subtilis* cells can compact their chromosome in response to antibiotic exposure, although it was drug specific, suggesting this response is conserved between the Actinobacteria and Firmicutes.

### 3.2 HBsu acetylation regulates nucleoid compaction in response to drug challenge

**Figure 2:**
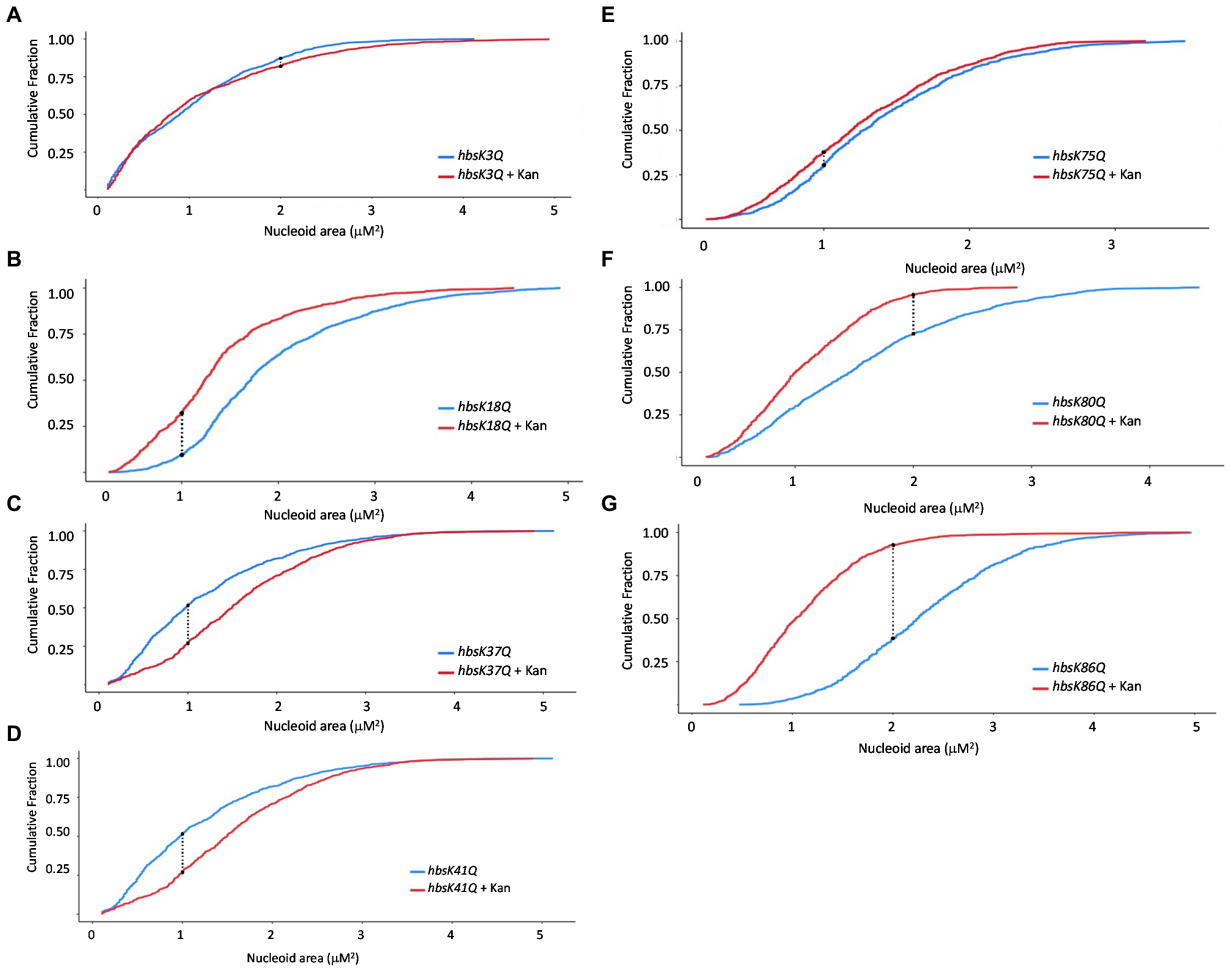
The impact of HBsu acetylation on nucleoid compaction following kanamycin exposure. *hbsK3Q* (BD8577, **A**), *hbsK18Q* (BD8219, **B**), *hbsK37Q* (BD8119, **C**), *hbsK41Q* (BD8147, **D**), *hbsK75Q* (BD8398, **E**), *hbsK80Q* (BD8576, **F**), and *hbsK86Q* (BD7493, **G**) cells were pre-grown in LB media for 2 h, then incubated with or without 5 μg/mL kanamycin for 20 min, and nucleoids stained with DAPI. Cumulative distribution plots are displayed, where the 50th percentile represents the median of the population distribution. Nucleoid areas of at least 800 cells were analyzed. The population distributions of nucleoid area in all strains, except for *hbsK3Q* (*p* value = 0.0682), were significantly different ± kanamycin, with the *hbsK75Q p* value *=* 0.005 and for all others, *p* values <2.2e^−16^.

Since wild-type cells compacted their nucleoids in response to drug challenge, we reasoned that this was likely due to HBsu, as it is known to be involved in DNA compaction (Micka and Marahiel, 1992; Köhler and Marahiel, 1997; Schibany et al., 2020) and is the predominant NAP in this *B. subtilis* (Ohniwa et al., 2011). In addition, we previously showed that lysine acetylation at key sites in HBsu regulates nucleoid compaction in cells grown in minimal media (Carabetta et al., 2019). With this in mind, we next explored the role of lysine acetylation of HBsu during drug challenge. For these analyses, we utilized a collection of strains in which the lysine acetylation site (K3, K18, K37, K41, K75, K80, and K86) was mutated to glutamine (K → Q) or arginine (K → R) to mimic the fully acetylated or deacetylated state, respectively (Carabetta et al., 2019). Cells were grown in the presence and absence of kanamycin for 20 min and nucleoids stained with DAPI for visualization and quantification. A time of 20 min was selected to observe early responses to antibiotic challenge. Compared to wild-type cells, *hbsK3Q* and *hbsK75Q* did not compact their nucleoids properly in response to kanamycin (compare Figures 2A,E to Figure 1B), suggesting that some level of deacetylation of these sites is required for this response. For the *hbsK18Q*, *hbsK80Q*, and *hbsK86Q* strains, there was a response similar to wild-type, where the nucleoid became compacted in the presence of kanamycin (Figures 2B,F,G). Note that in LB media, some of the Q mutants may lead to global decondensation of the chromosome in the absence of drug, such as *hbsK86Q*, making the response to drug seem more extreme than that seen with wild-type (compare Figure 2G with Figure 1B). Surprisingly, the *hbsK37Q* and *hbsK41Q* strains had an opposite response, in that the nucleoid was decondensed in response to kanamycin challenge (Figures 2C,D). This suggested that acetylation of these two sites relaxes the chromosome. The fact that they have the opposite response suggested that acetylation of K37 or K41 may be required to decondense the chromosome to escape from this drug-induced state.

**Figure 3:**
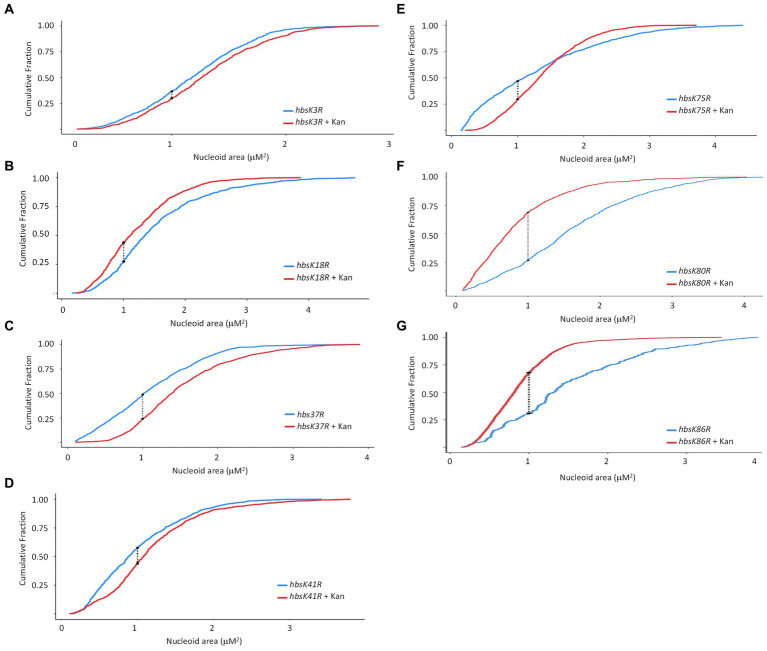
The impact of HBsu deacetylation on nucleoid compaction following kanamycin exposure. *hbsK3R* (BD8387, **A**), *hbsK18R* (BD8190, **B**), *hbsK37R* (BD8120, **C**), *hbsK41R* (BD8148, **D**), *hbsK75R* (BD8333, **E**), *hbsK80R* (BD7484, **F**), and *hbsK86R* (BD7506, **G**) cells were pre-grown in LB media for 2 h, then incubated with or without 5 μg/mL kanamycin for 20 min, and nucleoids stained with DAPI. Cumulative distribution plots are displayed, where the 50th percentile represents the median of the population distribution. The population distributions of nucleoid area in all strains were significantly different ± kanamycin: *hbsK3R* (*p* value = 0.005), *hbsK18R* (*p* value = 3.16e^−12^), *hbsK37R* (*p* value < 2.2e^−16^), *hbsK41R* (*p* value < 1.1e^−15^), *hbsK75R* (*p* value < 2.2e^−16^), *hbsK80R* (*p* value < 2.2e^−16^), and *hbsK86R* (*p* value < 2.2e^−16^).

In an *hbsK3R* and *hbsK41R* mutant, there were very small differences visually with or without kanamycin (Figures 3A,D), although by statistical analyses, the distributions were significantly different. However, these subtle changes in distribution are unlikely to be biologically significant. The nucleoids of these mutants were already compacted compared to wild-type (Figure 1B), so there were minor differences with and without drug. This supports that acetylation at K41 may be required to escape from the compacted state. For K3, the fully acetylated and deacetylated forms did not have an appropriate response, suggesting that some intermediate level of acetylation is required *in vivo*. For the *hbsK18R, hbsK75R*, *hbsK80R*, and *hbsK86R* mutants there was still a response where the nucleoids were more compacted in the presence of kanamycin (Figures 3B,E–G). Taken together, this suggests that the acetylation status of K18, K80, and K86 is not important for nucleoid compaction in the context of antibiotic challenge, and K75 must be deacetylated for this response. The *hbsK37R* mutant behaved exactly like the *hbsK37Q* mutant, where the nucleoid was decondensed when faced with antibiotic challenge. As K37 is not predicted to directly contact the DNA (Carabetta et al., 2019), perhaps mutation of this residue to any amino acid disrupts an important regulatory protein–protein interaction.

### 3.3 HBsu acetylation influences the rate of killing during antibiotic exposure

**Figure 4:**
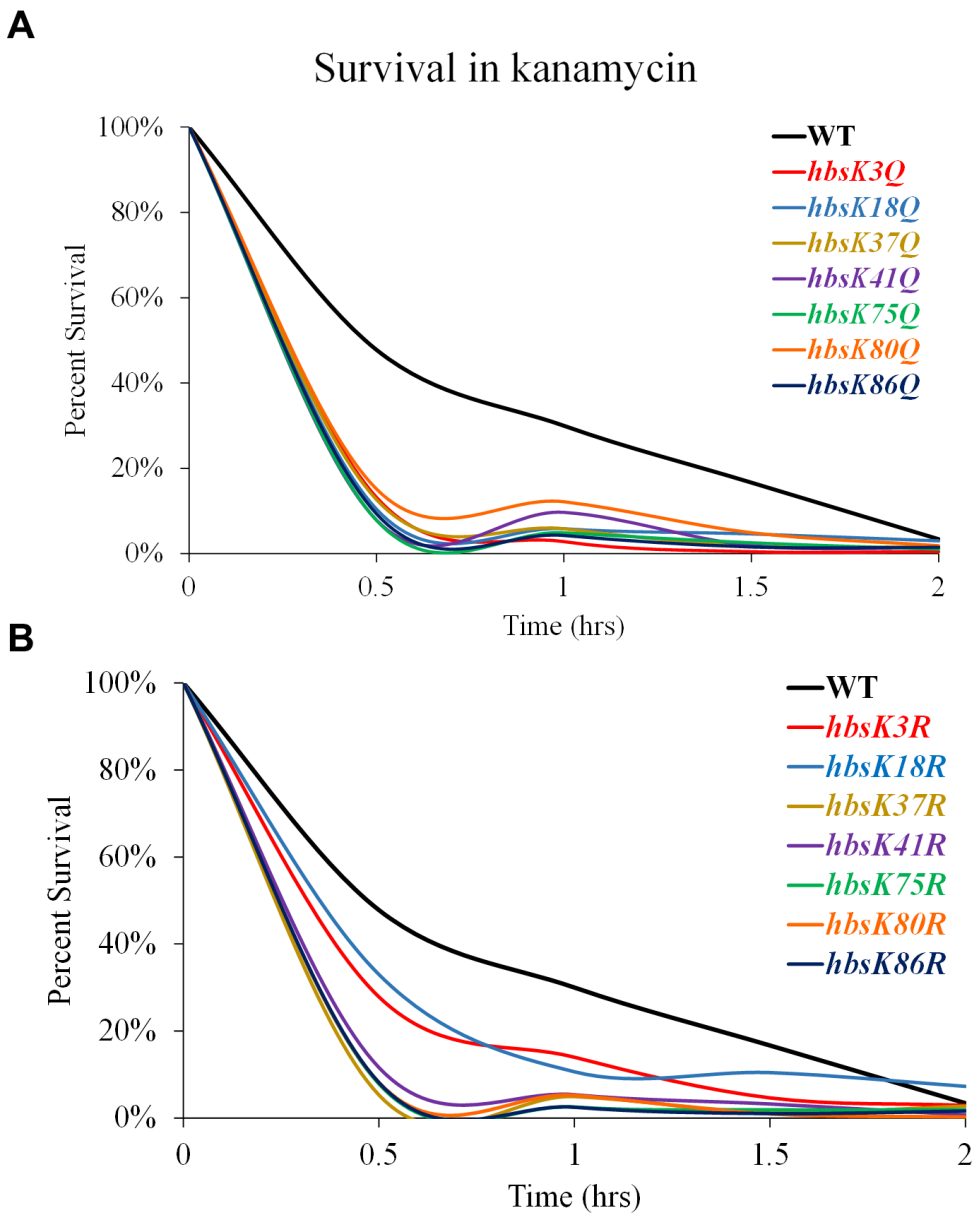
Interfering with HBsu acetylation leads to faster killing in the presence of kanamycin. Cells were pre-grown in LB media, then incubated with or without 5 μg/mL kanamycin for 2 h, with viable counts determined every 30 min. Percent survival at each timepoint was determined as CFUs/mL following kanamycin treatment/initial colony counts (CFUs/mL). **(A)** Wildtype (BD630), *hbsK3Q* (BD8577), *hbsK18Q* (BD8219), *hbsK37Q* (BD8119), *hbsK41Q* (BD8147), *hbsK75Q* (BD8398), *hbsK80Q* (BD8576), and *hbsK86Q* (BD7493). **(B)** Wildtype (BD630), *hbsK3R* (BD8387), *hbsK18R* (BD8190), *hbsK37R* (BD8120), *hbsK41R* (BD8148), *hbsK75R* (BD8333), *hbsK80R* (BD7484), and *hbsK86R* (BD7506).

**Figure 5:**
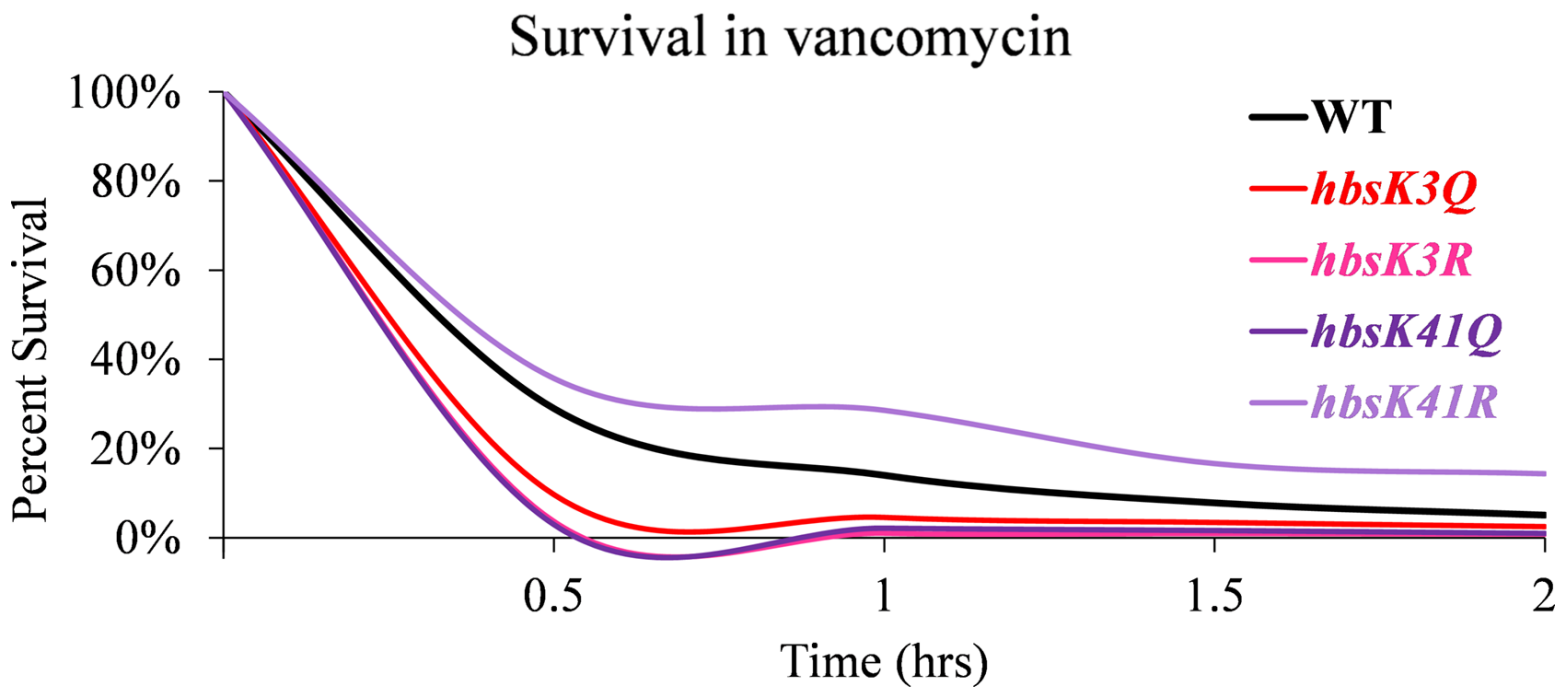
Interfering with HBsu acetylation leads to faster killing in the presence of vancomycin. Cells were pre-grown in LB media, then incubated with or without 12.5 μg/mL vancomycin for 2 h, with viable counts determined every 30 min. Percent survival at each timepoint was determined as CFUs/mL following vancomycin treatment/initial colony counts (CFUs/mL). Wildtype (BD630), *hbsK3Q* (BD8577), *hbsK41Q* (BD8147), *hbsK3R* (BD8387), and *hbsK41R* (BD8148).

As we observed that some mutants do not properly compact their nucleoids, we next determined if this observation impacted cell survival during antibiotic challenge. Mutant cells were grown overnight and diluted into fresh media with and without kanamycin, after which cell survival was analyzed every 30 min for 2 h. For wild-type cells, ~50% of the population was killed by 30 min of drug exposure, which steadily decreased over 2 h, ending with less than 5% survival (Figure 4; Supplementary Table S1). For the Q substitution mutants, all strains had a faster killing rate, especially in the first 30 min, when compared to wild-type cells, regardless of whether their nucleoids were properly compacted (Figure 4A). The percentage of survival decreased below 15% within 30 min of exposure to kanamycin. By one 1.5 h, the survival of all mutants was less than 5% (Supplementary Table S1). This suggested that the level of nucleoid compaction alone does not impact the rate of killing or overall survival of the population. Most of the R substitution mutants followed a similar pattern, with survivability decreased below 15% within 30 min of exposure to kanamycin (Figure 4B; Supplementary Table S2). The *hbsK3R* and *hbsK18R* mutants had an intermediate phenotype, where it took 1 h to decrease the population numbers below 15%. At the 2-h time point, for all strains there was on average 0.5–3.4% survival, which likely represents the viable persistent subpopulation. Next, we selected the *hbsK3Q* strain, which did not properly compact the nucleoid, and the *hbsK41Q* strain, which had expanded nucleoids in the presence of kanamycin, to analyze the survival kinetics following vancomycin exposure. As seen with kanamycin, the *hbsK3Q* and the *hbsK3R* strains displayed faster killing kinetics than the wild-type following vancomycin exposure (Figure 5; Supplementary Table S3). The *hbsK41Q* had faster kinetics as well, but the *hbsK41R* strain looked like wild-type and actually had a 2–3-fold increase in percentage of survivors at the later timepoints (Supplementary Table S3). The reason for this difference compared to kanamycin exposure is unclear but does suggest that it would be beneficial for cells to have K41 deacetylated when challenged with drugs. These findings confirm that the extent of nucleoid compaction does not influence the rate of killing following exposure to bactericidal drugs.

**Figure 6:**
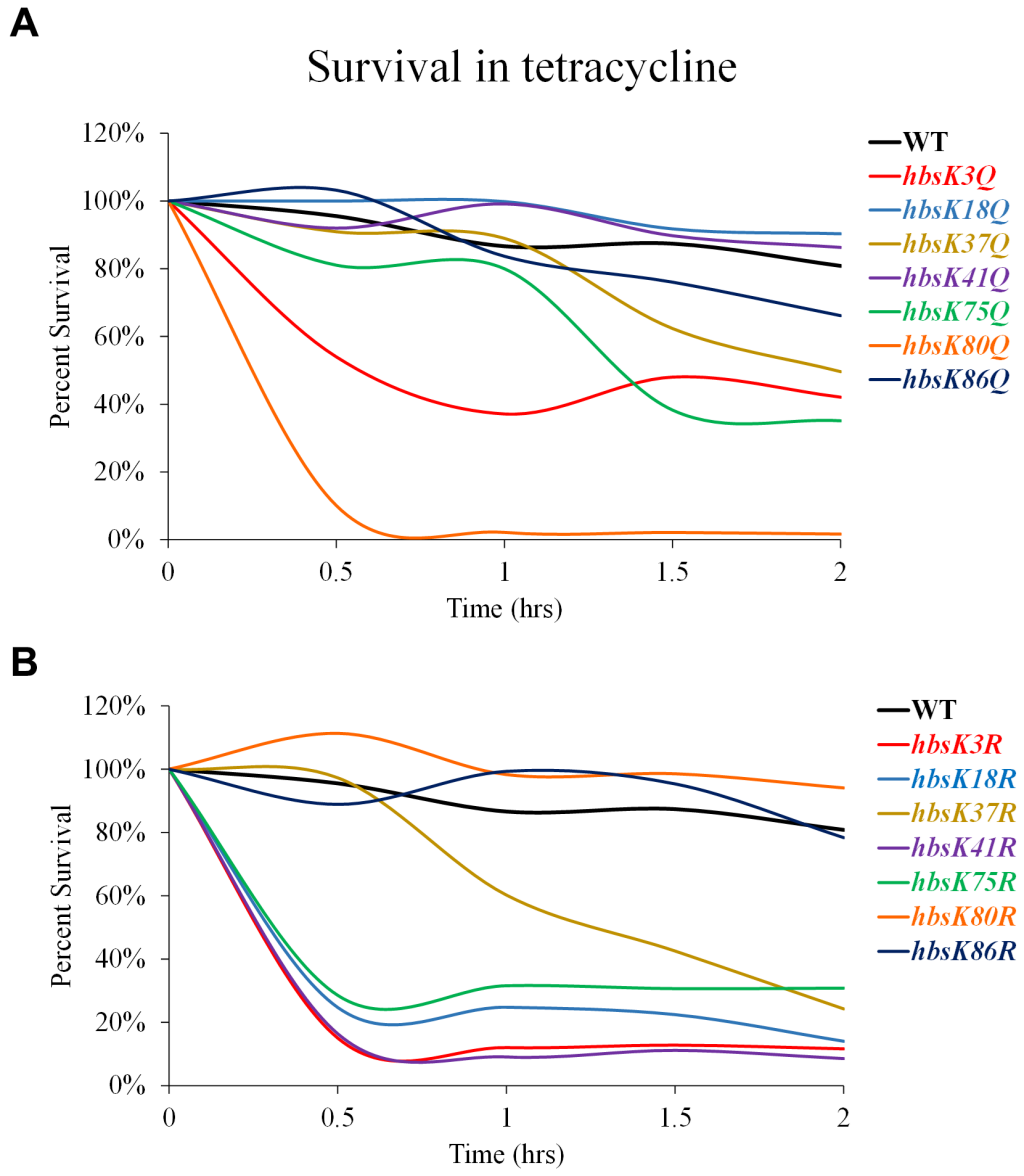
Interfering with HBsu acetylation alters survival in the presence of tetracycline. Cells were pre-grown in LB media, then incubated with or without 25 μg/mL tetracycline for 2 h, with viable counts determined every 30 min. Percent survival at each timepoint was determined as CFUs/mL following tetracycline treatment/initial colony counts (CFUs/mL). **(A)** Wildtype (BD630), *hbsK3Q* (BD8577), *hbsK18Q* (BD8219), *hbsK37Q* (BD8119), *hbsK41Q* (BD8147), *hbsK75Q* (BD8398), *hbsK80Q* (BD8576), and *hbsK86Q* (BD7493). **(B)** Wildtype (BD630), *hbsK3R* (BD8387), *hbsK18R* (BD8190), *hbsK37R* (BD8120), *hbsK41R* (BD8148), *hbsK75R* (BD8333), *hbsK80R* (BD7484), and *hbsK86R* (BD7506).

We also examined the survival of these mutant strains against the bacteriostatic drug tetracycline (Figure 6). At 2 h, there was only a 20% reduction in survival, which was variable among replicates, in viability for wild-type cells (Figure 6; Supplementary Table S4). The *hbsK18Q, hbsK41Q*, and *hbsK86Q* mutants were relatively similar to wild-type (Figure 6A; Supplementary Table S4). The *hbsK37Q* displayed an increased rate of killing after 1 h of exposure, whereas *hbsK3Q, hbsK75Q,* and *hbsK80Q* were rapidly killed, with *hbsK80Q* being the most severe. The opposite mutant, *hbsK80R* followed a similar pattern to the wild-type, suggesting that in wild-type cells, K80 is mostly deacetylated (Figure 6B, Supplementary Table S5). In addition, K18 is normally acetylated, as *hbsK18Q* cells were similar to wild-type, while the *hbsK18R* mutant cells were rapidly killed (Figure 6). The *hbsK86R* mutant was like wild-type, while all other mutants were rapidly killed in the presence of tetracycline. It is unknown whether the response to bacteriostatic and bactericidal drugs is the same, but our data suggests that HBsu may be important for more than nucleoid compaction and that acetylation influences these functions differently.

### 3.4 The effects of HBsu acetylation on the formation of persistent cells

**Figure 7:**
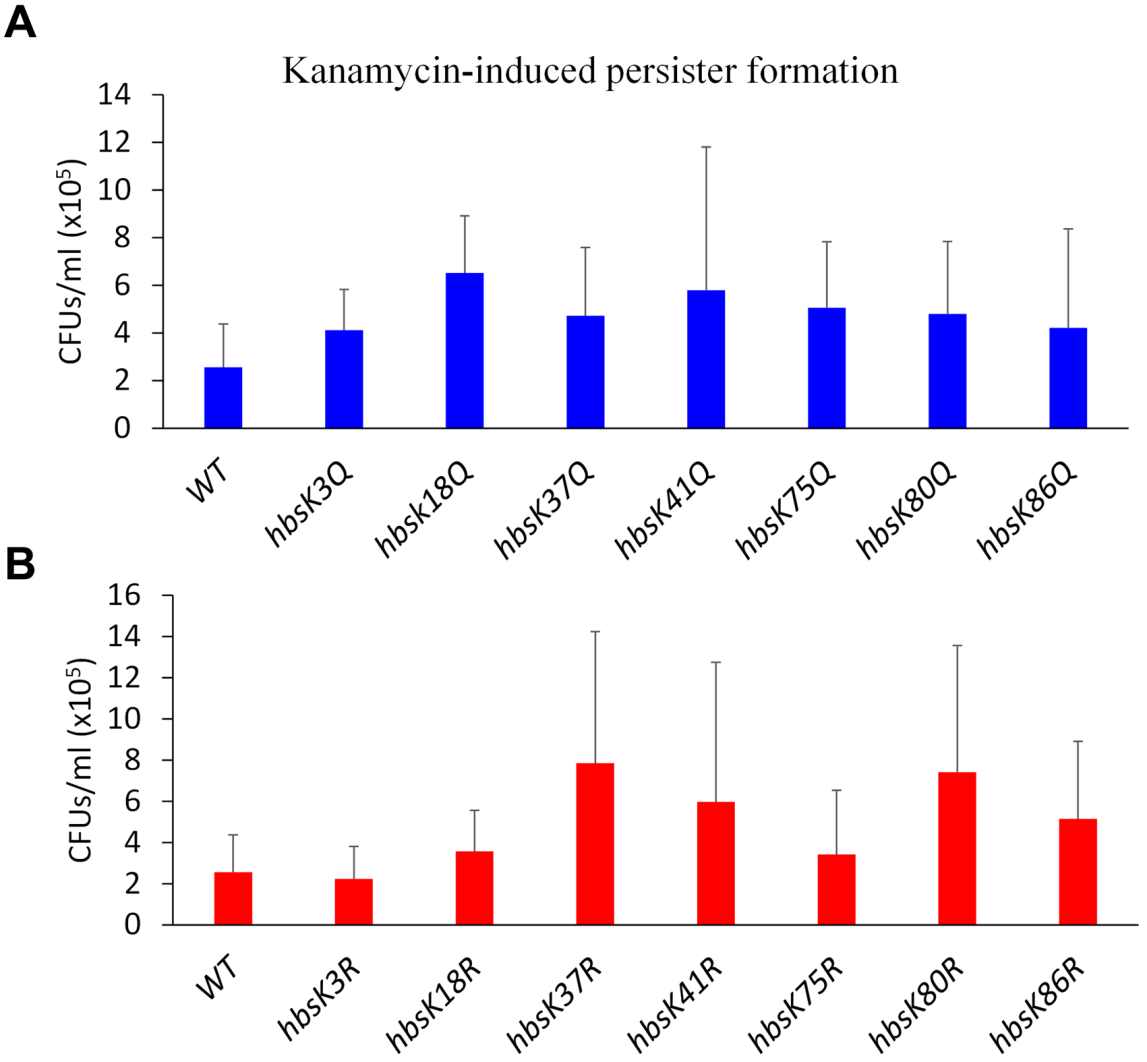
The influence of HBsu acetylation the formation of persisters. Cells were pre-grown in LB media for 1 h, and then exposed to 5 μg/mL kanamycin for 5 h. Serial dilutions were plated to determine survivors (CFUs/mL), which likely represented persisters. The data shown is an average of at least three independent replicates with standard deviation displayed. **(A)** Wildtype (BD630), *hbsK3Q* (BD8577), *hbsK18Q* (BD8219), *hbsK37Q* (BD8119), *hbsK41Q* (BD8147), *hbsK75Q* (BD8398), *hbsK80Q* (BD8576), and *hbsK86Q* (BD7493). **(B)** Wild-type (BD630), *hbsK3R* (BD8387), *hbsK18R* (BD8190), *hbsK37R* (BD8120), *hbsK41R* (BD8148), *hbsK75R* (BD8333), *hbsK80R* (BD7484), and *hbsK86R* (BD7506). CFU, Colony forming unit; WT, Wildtype.

So far, our data suggested that the acetylation of HBsu impacts survival during antibiotic stress. One explanation is that acetylation regulates DNA compaction, which we have provided evidence for above, and another possibility that HBsu regulates gene expression and the development of antibiotic-tolerant subpopulations, as found for the HBsu ortholog in *M. smegmatis* (Sakatos et al., 2018). To test this idea, wild-type and mutant cells were pre-grown in LB and subsequently exposed to kanamycin for 5 h before plating to assess survivors, which represent the persistent population. In comparison to wild-type, the *hbsK18Q* mutant exhibited a 2.55-fold increase in persisters, the *hbsK75Q* mutant showed a 1.98-fold increase, and the *hbsK41Q* mutant had a 2.26-fold increase, while all other Q mutants were only modestly increased compared to wild-type (Figure 7A). The *hbsK41R* mutant showed a similar increase to the *hbsK41Q* mutant of 2.34-fold. For the remaining R mutants, *hbsK37R* showed a 3-fold increase, *hbsK86R* had a 2-fold increase, and *hbsK80R* exhibited a 2.9-fold increase (Figure 7B). It should be noted that our data were highly variable among replicates, so we cannot draw strong conclusions. However, if we accept that the patterns were consistent among replicates, then this data suggested that in wild-type cells, K18 and K75 are deacetylated, while K37, K80, and K86 are acetylated in persistent cells. The acetylation status at K41 was unclear, but there is likely some level of acetylation required. Perhaps, cells with this acetylation pattern are more likely to enter the persistent state, which is based upon which genes are expressed. Understanding HBsu acetylation and its consequent effects is important for determining the specific role of acetylation and its connection to bacterial persistence which holds implications for developing strategies to regulate persistence in clinically relevant bacteria.

### 3.5 HBsu acetylation is required for recovery from the persistent state

**Figure 8:**
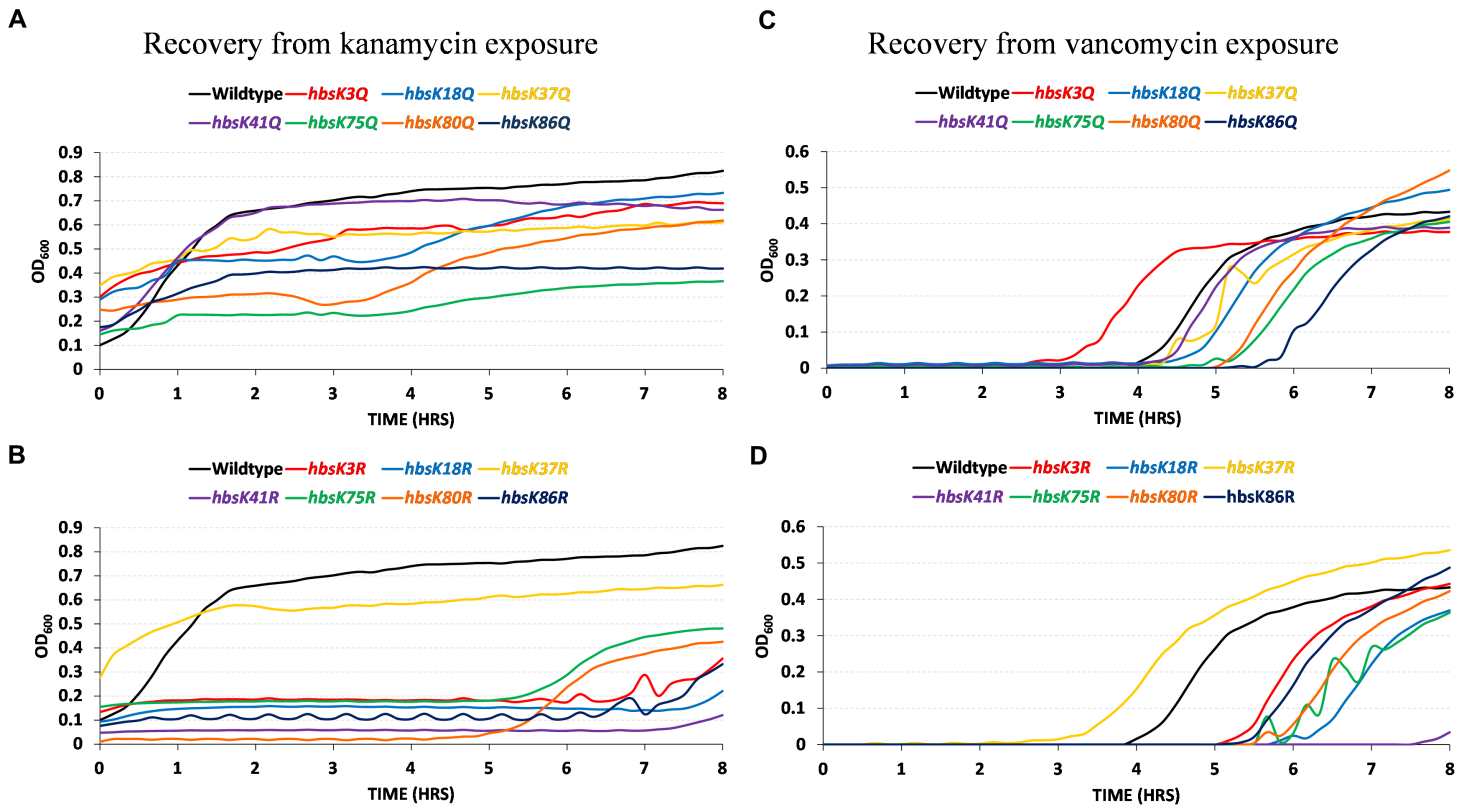
HBsu acetylation influences recovery from the persistent state. Persistent cells were prepared in the presence of kanamycin **(A,B)** and vancomycin **(C,D)**, as described in the Materials and Methods section. Cell pellets were washed three times in LB without antibiotics and added to a 96-well plate. Growth was monitored by OD_600_ determination every 10 min for 8 h in a microplate reader. The curves plotted are the averages of six determinations (three technical replicates for each of two biological replicates). **(A,C)** Wild-type (BD630), *hbsK3Q* (BD8577), *hbsK18Q* (BD8219), *hbsK37Q* (BD8119), *hbsK41Q* (BD8147), *hbsK75Q* (BD8398), *hbsK80Q* (BD8576), and *hbsK86Q* (BD7493). **(B,D)** Wild-type (BD630), *hbsK3R* (BD8387), *hbsK18R* (BD8190), *hbsK37R* (BD8120), *hbsK41R* (BD8148), *hbsK75R* (BD8333), *hbsK80R* (BD7484), and *hbsK86R* (BD7506).

While the overall numbers of persisters were increased in the presence of altered HBsu acetylation, we next determined if the cells that survived could properly escape from the semi-dormant, persistent state. Following the formation of persister cells in the presence of kanamycin and vancomycin, cells were washed and diluted into media without drugs and monitored for growth for 8 h in a multimode plate reader. The persisters from wild-type cells in the presence of kanamycin began growing within the first 30 min following dilution into fresh media (Figure 8A). The same was observed for *hbsK41Q* mutant cells. For all the other Q mutants, the persisters did grow, but at a slower rate than wild-type. The slowest growth rate was observed for the *hbsK75Q* strain (Figure 8A). For the deacetylated mimics, *hbsK37R* was similar to wild-type, while all the other mutants delayed growth significantly (Figure 8B). The *hbsK80R* and *hbsK75R* strains did not resume growth until after 5 h, while the *hbsK3R* and *hbsK86R* strains resumed growth between 6 and 7 h. The *hbsK18R* and *hbsK41R* strains resumed growth close to 8 h. As the *hbsK37R* strain did not delay growth and is the only one of the seven that does not have nucleoid compaction phenotype (Carabetta et al., 2019), this data suggested that nucleoid compaction is necessary to enter the persistent state and decompaction is required for escape.

Similar observations were made when the persister cells were harvested from vancomycin-treated cultures. Wild-type cells began growing after a 4-h delay (Figure 8C). The *hbsK3Q* strain began growing an hour earlier, while the *hbsK18Q*, *hbsK37Q*, *hbsK41Q*, *hbsK75Q*, and *hbsK80Q* strains began growth between 4 and 5 h. The *hbsK86Q* strain was the most delayed, beginning to grow at 5.5 h. For the opposite mutants, the *hbsK37R* strain began growth earlier than wild-type, but all other R mutants were delayed, beginning to grow after 5 h (Figure 8D). The largest defect was observed with the *hbsK41R* mutant, with growth only being observed close to 8 h. Together, these findings support that idea that K41 must be acetylated for decompaction of the chromosome to occur, as in either vancomycin or kanamycin, the *hbsK41R* mutants, had a severe phenotype.

## 4 Discussion

DNA compaction is widely acknowledged as an effective strategy employed by bacteria in response to environmental stressors (Boor, 2006; Hołówka and Zakrzewska-Czerwińska, 2020). Given the diverse environments that bacteria inhabit, ranging from extreme heat to cold or nutrient-rich to poor, DNA compaction is a rapid and practical mechanism to protect the chromosome from insults and enhance survival chances. Compaction of the nucleoid is crucial not only for spatial organization but also efficient segregation of replicated DNA during cell division, regulation of bacterial responses to environmental changes, and gene expression. Under stressful conditions, DNA compaction not only provides heightened protection but also affords bacteria the ability to conserve space and energy. This streamlined energy usage becomes crucial when bacteria must redirect their resources more efficiently, prioritizing tasks such as maintenance and response to stresses. *Escherichia coli, Helicobacter pylori, Deinococcus radiodurans*, and *B. subtilis* transiently condense their chromosomal DNA in response to different stresses to preserve genome integrity (Wolf et al., 1999; Smith et al., 2002; Eltsov and Dubochet, 2005; Ceci et al., 2007). Furthermore, recently, it was shown *M. tuberculosis* condenses the chromosome in response to the stressors of nutrient starvation and antibiotic treatment, and this response was important to survival (Scutigliani et al., 2018). However, it was unclear whether this response to antibiotic challenge represented a universal survival strategy in bacteria. Here, we showed that wild-type cells, when challenged with kanamycin, compacted the nucleoid as compared to cells not challenged (Figure 1). This suggested that nucleoid compaction may also be a bacterial survival strategy when challenged with antibiotics, perhaps to protect the DNA from drug-induced oxidative damage. Aminoglycosides target the bacterial ribosome but are also thought to induce the production of ROS, which is part of their killing mechanism (Kohanski et al., 2008). However, wild-type cells did not compact the nucleoid when challenged with tetracycline or vancomycin (Supplementary Figure S1). Tetracyclines and other bacteriostatic drugs do not lead to the production of hydroxyl radicals (Kohanski et al., 2007). In *Staphylococcus aureus*, exposure to vancomycin did lead to the production of hydroxyl radicals but following 3 h of treatment (Kohanski et al., 2007). For our microscopy studies, we examined cells after 20 min of exposure, to look for early responses and to ensure adequate cell numbers for quantification (Figure 1; Supplementary Figure S1). We propose that nucleoid compaction is a response to antibiotic-induced oxidative stress, and we predict that examination of cells following prolonged exposure to vancomycin would result in compacted nucleoids. The identity of the protein(s) that senses the stress and signals to condense the chromosome is currently unknown. We propose that one signal is HBsu acetylation, and then the signaling proteins would be those enzymes that modify HBsu, or other histone-like proteins in other species. With candidate enzymes identified, these ideas can be further explored. Additionally, further studies are required to confirm if chromosomal condensation is a response only to bactericidal drugs that induce oxidative stress or DNA damage (Kohanski et al., 2007).

**Figure 9:**
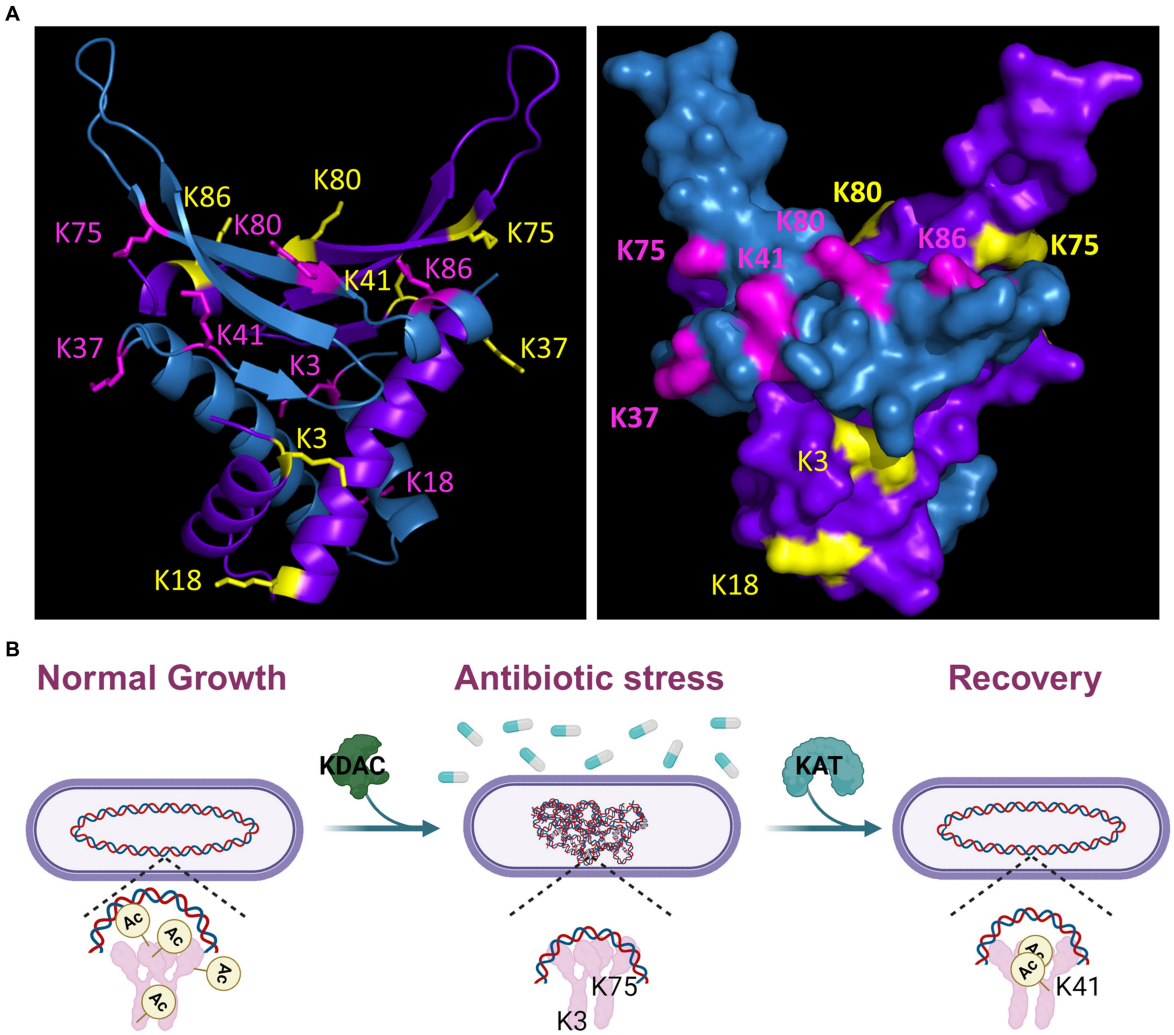
Model of HBsu response to antibiotic stress. **(A)** A *B. subtilis* HBsu structural model was generated in PyMOL using the *B. stearothermophilus* ortholog as a template (PDB 1HUU). The HBsu homodimer is displayed, with one monomer colored in cyan with the acetylated lysine sites (K3, K18, K37, K41, K75, K80, and K86) labeled in pink, and the other monomer colored purple with the acetylated sites labeled yellow. The left panel is a ribbon diagram, and the right shows a space-filling model. Reproduced and modified with permission from Carabetta et al. (2019). **(B)** During normal, exponential phase growth, HBsu is acetylated in specific patterns over the chromosome (left). In response to antibiotic stress, possibly only those that induce the formation of ROS, HBsu is deacetylated by an unknown KDAC, especially important at K3 and K75, which leads to a more compacted nucleoid (middle). This compaction may protect against further damage and/or change the transcriptional program to aid in survival. When the stress is removed, an unknown KAT acetylates HBsu and acetylation at K41 is likely an important early event to trigger the reentry into growth (right).

The NAPs are the likely drivers of the DNA compaction response, as they are responsible for chromosome dynamics and organization. In addition, it is well established that they are important for adaptation to unfavorable conditions, including sudden stressors (Atlung and Ingmer, 1997; Nguyen et al., 2009; Mangan et al., 2011; Datta et al., 2019; Hołówka and Zakrzewska-Czerwińska, 2020). For both *M. tuberculosis* and *B. subtilis*, acetylation of the HU family proteins regulates DNA compaction. Here, we showed that mutation of specific acetylation sites of HBsu eliminates the nucleoid compaction response to drug challenge (Figure 2). Based on our findings, some level of acetylation at K3 was required for a proper response, and deacetylation at K75 was important. Acetylation at sites K18 and K80 were unimportant for nucleoid compaction under these conditions. For *hbsK41R* there were very small differences in the presence of absence of kanamycin, which should suggest that K41 must be acetylated (Figure 3). However, the *hbsK41Q* mutant resulted in a rightward shift, toward larger, expanded nucleoids. While this was unexpected, we propose that acetylation at K41 is required to decondense the chromosome to escape from this drug-induced state. This suggestion was supported by our data that showed that *hbsK41R* persisters from kanamycin or vancomycin-treated cells significantly delayed the entry into growth following removal of the antibiotic stress (Figures 8B,D). Another unusual observation was that both K37 mutants had distribution curves that were right shifted in the presence of drug. K37 was the only site where mutation to arginine did not lead to a compacted nucleoid in minimal media (Carabetta et al., 2019). The K37 persister cells escaped from the persistent state similar or earlier than wild-type (Figure 8), further suggesting that a relaxed chromosome is needed to escape the persistent state. K37 is not predicted to make direct contact with the chromosome (Figure 9). In this model, the DNA molecule is threaded through the extended “arms” on top of HBsu, and likely wraps down around it, which induces a severe bend in the DNA molecule that compacts and organizes the chromosome (Swinger and Rice, 2007). K37 points in the opposite direction of where the DNA molecule lies. It seems likely that the acetylation status at K37 does not influence chromosomal dynamics by direct DNA binding. Instead, we propose that this is an important interface for protein–protein interactions, and introduction of a non-native amino acid abolishes this interaction. By this model, the unknown protein would be regulatory in nature, in that it modulates the DNA binding activity of HBsu, possibly through acetylation of the other sites. It will be interesting to study the influence of the known HBsu acetyltransferases, YfmK and YdgE (Carabetta et al., 2019), and examine the possible contribution of the two known deacetylases in *B. subtilis,* AcuC and SrtN (Gardner and Escalante-Semerena, 2009). In addition, little is known about how these enzymes themselves are regulated and it is worth determining which one(s) are responsible setting the proper acetylation pattern on HBsu during times of stress.

In *M. tuberculosis*, the lack of nucleoid condensation is associated with decreased survival and recovery from the persistent state (Scutigliani et al., 2018). Interestingly, nearly all mutants tested displayed increased rates of killing in the presence of kanamycin and vancomycin (Figures 4, 5). The faster rate of death observed in the mutants could be due to the lack of significant compaction of the nucleoid for the *hbsK3Q and hbsK75Q* mutants. Nucleoid compaction is a protective response, whereby NAPs bind along the entire chromosome, compact the chromosome, and protect it from insults (Stojkova et al., 2018; Hołówka and Zakrzewska-Czerwińska, 2020). The DNA compaction response is also important for damage repair, such as double-stranded break repair (Shechter et al., 2013) and DNA replication initiation (Karaboja and Wang, 2022), which could also be the underlying reason for increased death rates. These possibilities are not mutually exclusive and further studies will be required to determine the exact underlying mechanism. Nonetheless, acetylation at K3 and K75 likely leads to a significant reduction in DNA binding affinity, as these sites likely contact the DNA molecule (Figure 9), thus making chromosome compaction less efficient or not effective. However, strains that did properly compact their nucleoids also exhibited faster death rates. As acetylation could influence multiple aspects of HBsu functions, other possibilities for reduced survival are that there is improper stress response signaling, or changes in gene expression of proteins that promote survival. Indeed, the *E. coli* HU orthologs, HupA and HupB were found to control gene expression, where the regulon consists of ~350 genes, including many involved in stress responses (Oberto et al., 2009). Note that the regulon of HBsu is currently unknown and future investigations should be performed to determine if the acetylation status of HBsu alters gene expression during times of stress.

In *M. smegmatis*, it was found that acetylation of HupB regulates the emergence of a specific slow-growing, drug-tolerant subpopulation, likely by controlling gene expression (Sakatos et al., 2018). Specifically, mutation of K86, which is modified by both acetylation and methylation in this species, to the unmodified mimic resulted in a loss of the SCV subpopulation. Here, we found that acetylation of HBsu at K18, K41, and K75 led to at least 2-fold increases in the number of persisters (Figure 7A). This would be predicted based upon the data from *M. smegmatis*. Surprisingly, we did not see decreases in persisters in any mutants but observed increased numbers for some of the R mutants, specifically at K37, K41, K80, and K86 (Figure 7B). It is unclear if SCVs or LCVs exist in *B. subtilis*, so this could be one explanation for the observed differences. Although our data was highly variable, there was a suggestion that the acetylation status of HBsu influences gene expression, making the transition into the persistent state more likely. However, once in the persistent state, altering the acetylation status of HBsu makes the escape more challenging (Figure 8). Our data suggests that acetylation of HBsu is required to escape the persistent state, with K41 possibly being the most important site. All the R mutants, except for *hbsK37R* were delayed in re-entering growth, and we propose that this is because the nucleoids were highly compacted, which did allow for the resumption of DNA replication or transcription due to lack of access to the chromosome. In addition, our data suggests that while DNA compaction may be an important response as an attempt to maintain genome integrity, it is of most benefit to the persistent population. A more in-depth transcriptomic analysis of these persisters is warranted to fully understand the influence of HBsu acetylation on persister cell formation and survival.

The persistent subpopulation is what makes pathogens able to remain dormant and survive antibiotic challenge in patients. Initially thought to be rare, persister cells are non-growing cells capable of withstanding drug concentrations many times higher than the minimum inhibitory concentration (Bigger, 1944; Sakatos et al., 2018). The clinical implication of bacterial persistence is treatment failure, which can result in chronic infections. The persisters represent only a subpopulation, meaning that while the majority of the infection will be killed by antibiotics, the persisters survive. When the antibiotic stress is removed, i.e., due to the completion of a regimen, the persisters can grow and divide again, leading to recurrent infections. In addition, when persisters survive the initial antibiotic exposure, there is an increased likelihood of acquiring resistance through genetic mutation or HGT, possibly from interactions with the native microbiota (Huemer et al., 2020). Alarmingly, this dual threat not only allows persisters to survive standard treatment, but also contributes to the emergence of more resistant bacterial strains, limiting the effectiveness of available antibiotics.

As chronic infections are a significant healthcare burden and increase morbidity and mortality for patients, it would be worthwhile to further explore how modulation of the acetylation status of the histone-like proteins influences drug-tolerant sub-populations in both Gram-negative and -positive bacteria. If it is found that interfering with DNA condensation or gene expression reduces survival or eliminates drug-tolerant subpopulations, the design of novel drugs that block or interfere with histone-like protein acetylation could be pursued. In fact, in *M. smegmatis,* the acetyltransferase Eis acetylates HupB, which limits its DNA-binding capability. Scutigliani et al. (2018) showed that treatment of *M. smegmatis* with fusidic acid to induce DNA condensation, followed by Eis inhibitors led to drug synergy and more efficient killing. They proposed that by inhibiting acetylation, the nucleoid remained compacted, and the cells could not escape from the dormant persistent state (Scutigliani et al., 2018). We observed that some acetylation mutants in combination with tetracycline displayed synergistic effects, leading to rapid killing in the presence of a bacteriostatic drug. There have been previous reports of tetracyclines having synergistic effects and enhancing the potency of other drugs (Mawabo et al., 2015; Cacace et al., 2023). If we consider the HBsu mutations as a phenotypic mimic of a hypothetical drug that interferes with chromosomal compaction or organization, the combination of this hypothetical drug with tetracyclines might represent a novel strategy to augment and enhance treatment with bacteriostatic agents.

A drug that interferes with HU-family protein acetylation could potentially treat infections by killing actively growing cells and eliminating some persistent subpopulations, creating a more effective treatment option. Our findings suggest that acetylation of K41 is required to decondense the nucleoid and escape the persistent state (Figures 2, 3, 7, 8). If this finding is validated, identification of the appropriate acetyltransferase responsible for K41 acetylation during antibiotic stress could reveal a new target for novel drug design. Our lab has identified five potential enzymes (Carabetta et al., 2019). This drug could be used in an analogous fashion to those in *M. smegmatis*, where it may be more effective at killing in combination with aminoglycosides or tetracylcines. It is worth noting that in the Gram-positive pathogenic bacterium *S. aureus*, a recent report identified acetylation sites on the HBsu ortholog (Hsa) as K3, K18, K38, K67, K75, K80, K83, and K86 (Tan et al., 2022). Based on our data, if we assume chromosome compaction is a universal bacterial response to drug-induced stress, we predict that K38 acetylation of HSa plays a similar role as K41 acetylation of HBsu for recovery from a drug-induced, persistent state. If these predictions hold true, it is possible that we could identify broad-spectrum drug targets that can be used in the fight against drug-resistant infections.

## 5 Conclusion

As more largescale proteomic analyses of bacterial PTMs were published, it became clear that the HU-family of proteins were routinely identified among the modified proteins in diverse species (Carabetta, 2021). The HU-family of proteins is the most widely conserved NAP among bacteria, and this includes the conservation of the regulation by PTMs. In *B. subtilis,* we identified seven novel acetylation sites. So far, we know that some level of acetylation is required at six of those sites, all except for K37, for proper chromosomal compaction during growth (Carabetta et al., 2019). K37 is the only site that is not predicted to directly contact the DNA (Figure 9), which suggests that it may represent an important protein–protein interface, where acetylation can regulate this interaction. We also examined the impact of HBsu acetylation on sporulation and found that K41 acetylation is not important and deacetylation at K3 is required for normal sporulation frequency. For all other sites, some level of acetylation is needed. The patterns of acetylation required for resistance properties of mature spores were different (Luu et al., 2022). This data suggested that there may be sporulation-specific regulation of acetyltransferases or deacetylases that set the HBsu acetylation patterns, both before and after chromosomal packing into the mature spore. Here, we have expanded upon those findings. Our current model is that during exponential growth, HBsu is acetylated in specific, but currently unknown, patterns over the entire chromosome (Figure 9B). We propose that local areas on the chromosome contain highly acetylated HBsu species and these regions contain genes that are actively transcribed, analogous to eukaryotic histones. In response to antibiotic stress, possibly only those that induce the formation of ROS, HBsu is deacetylated by an unknown KDAC, which begins the compaction process. Our data suggests that K3 and K75 deacetylation is particularly important. This compaction may protect against further DNA damage. However, as most of the mutants displayed faster killing kinetics in the presence of drug, regardless of their nucleoid compaction state, we suggest that HBsu plays additional roles, possibly by altering the transcriptional program to ensure survival. When the stress is removed, an unknown KAT acetylates HBsu. Acetylation of K41 is particularly important and seems to be a signal to resume growth and decondense the chromosome. In the persistent subpopulation (Figure 7), K18 and K75 were deacetylated, while K37, K80, K86 were acetylated. Perhaps this acetylation pattern regulates gene expression or important protein–protein interactions and allows for cells to remain in a slow growing, metabolically inactive state. One thing is for certain, there are different acetylation patterns of HBsu that have phenotypic consequences depending on the growth conditions, which lends further support for the existence of a histone-like code in bacteria. The missing piece of information for all these studies is an understanding of how HBsu acetylation influences gene expression. Currently, we do not know where HBsu specifically binds on the chromosome during growth or under stress conditions, and we do not know the members of its regulon. This information must be determined next, using largescale omics studies, like RNA-seq, ChIP-seq, and Hi-C. With this information available, we can examine the effects of our collection of acetylation mutant strains to determine the influence of acetylation on gene expression during different developmental fates or during stressful conditions. If protein acetylation of HBsu influences the transition into the persistent state, recovery from persistence, or contributes to antibiotic survival, the identification of novel molecules that can inhibit this process, by targeting an acetyltransferase, deacetylase, or HBsu itself, could become new antimicrobial weapons against Gram-positive infections. A mechanism to target or eliminate the persistent population would tremendously reduce the risk of recurrent infections for patients and would lead to more efficient treatment. This promising and exiting possibility deserves further exploration.

## Data availability statement

The original contributions presented in the study are included in the article/Supplementary material, further inquiries can be directed to the corresponding author.

## Author contributions

RC: Validation, Visualization, Writing – original draft, Writing – review & editing, Formal analysis, Investigation, Project administration. TT: Formal analysis, Investigation, Writing – review & editing. PN: Formal analysis, Investigation, Writing – review & editing. LJ: Formal analysis, Investigation, Writing – original draft. KJ: Formal analysis, Investigation, Writing – original draft. DA: Formal analysis, Investigation, Writing – original draft. AK: Formal analysis, Investigation, Visualization, Writing – original draft. AP: Formal analysis, Investigation, Writing – review & editing. VC: Conceptualization, Funding acquisition, Resources, Supervision, Validation, Visualization, Writing – original draft, Writing – review & editing. BR: Investigation, Visualization, Writing – original draft, Writing – review & editing.
