# Supplementary material for: Nε-lysine acetylation of the histone-like protein HBsu influences antibiotic survival and persistence in Bacillus subtilis
Source: Front Microbiol. 2024 May 21;15:1356733. doi: 10.3389/fmicb.2024.1356733 (PMC11148388; doi:10.3389/fmicb.2024.1356733)
Supplement: Supplementary file 1 [file Data_Sheet_1.PDF]

Supplementary Material

N^{ε}-lysine acetylation of the histone-like protein HBsu influences antibiotic survival and persistence in *Bacillus subtilis*

Rachel A. Carr, Trichina Tucker, Precious M. Newman, Lama Jadalla, Kamayel Jaludi, Briana E. Reid, Damian N. Alpheus, Anish Korrapati, April Privonka, and Valerie J. Carabetta

Supplementary Figure S1

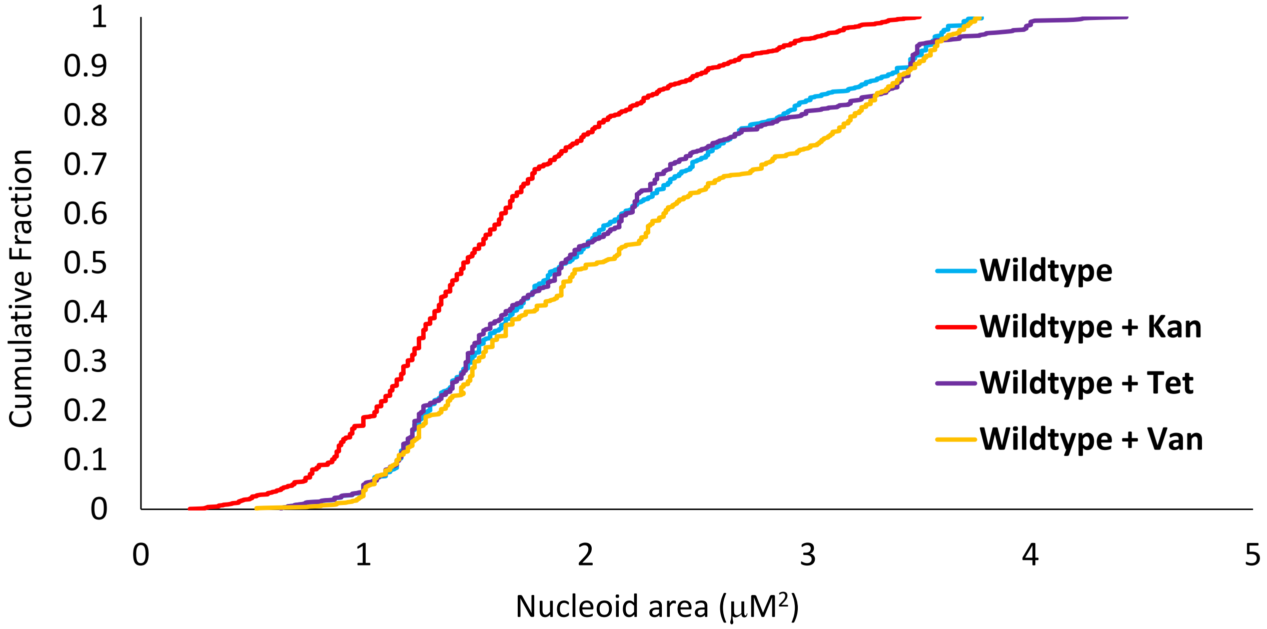

Figure S1: Wild-type cells compact their nucleoids in response to kanamycin, but not tetracycline or vancomycin. Wild-type cells (BD630) were pre-grown in LB media for 2 hrs, then incubated with or without 5 μg/ml kanamycin, 12.5 μg/ml vancomycin, or 25 μg/ml tetracycline for 20 minutes, and nucleoids stained with DAPI. Cumulative distribution plots are displayed, where the 50th percentile represents the median of the population distribution. Nucleoid areas of at least 800 cells were analyzed. The distributions with and without tetracycline were not significantly different (p-value= 0.6385), whereas the distribution with and without vancomycin was significantly different (p-value=0.0004). Statistical significance was determined by the Kolmogorov-Smirnov test.

Supplementary Table S1

	Average				
	0	0.5	1	1.5	2
WT	100%	47.8%	30.3%	11.5%	4.5%
hbsK3Q	100%	13.2%	4.3%	0.5%	0.5%
hbsK18Q	100%	10.4%	5.8%	4.6%	3.0%
hbsK37Q	100%	12.7%	5.9%	1.8%	1.6%
hbsK41Q	100%	7.9%	9.7%	1.9%	1.3%
hbsK75Q	100%	7.9%	4.5%	2.6%	1.3%
hbsK80Q	100%	15.2%	12.2%	4.9%	1.9%
hbsK86Q	100%	9.3%	4.3%	1.6%	1.5%

	Standard Deviation				
	0	0.5	1	1.5	2
WT	0%	55.0%	31.3%	18.4%	3.7%
hbsK3Q	0%	7.8%	1.2%	0.4%	0.4%
hbsK18Q	0%	1.6%	1.2%	3.6%	4.0%
hbsK37Q	0%	1.3%	4.0%	1.2%	1.1%
hbsK41Q	0%	4.2%	3.7%	1.6%	0.4%
hbsK75Q	0%	2.0%	3.5%	2.2%	1.1%
hbsK80Q	0%	12.4%	7.1%	2.7%	1.0%
hbsK86Q	0%	2.0%	2.7%	0.2%	0.2%

Average survival at each time point (0, 0.5, 1, 1.5 and 2 hours) of at least three replicates, corresponding to Figure 4A. The standard deviations for each strain and timepoint are displayed. WT: Wildtype.

Supplementary Table S2

	Average				
	0	0.5	1	1.5	2
WT	100%	47.8%	30.0%	16.7%	3.4%
hbsK3R	100%	27.8%	14.0%	4.6%	2.9%
hbsK18R	100%	33.0%	10.6%	10.4%	7.3%
hbsK37R	100%	5.3%	4.9%	1.3%	2.7%
hbsK41R	100%	11.6%	5.4%	3.2%	0.7%
hbsK75R	100%	7.8%	2.5%	1.9%	1.9%
hbsK80R	100%	8.2%	5.2%	0.9%	0.3%
hbsK86R	100%	8.1%	2.5%	1.0%	1.6%

	Standard Deviation				
	0	0.5	1	1.5	2
WT	0%	55.0%	31.3%	18.4%	3.7%
hbsK3R	0%	23.7%	4.6%	6.5%	4.2%
hbsK18R	0%	35.7%	7.5%	3.5%	0.5%
hbsK37R	0%	5.3%	5.9%	1.5%	2.9%
hbsK41R	0%	5.4%	3.4%	1.2%	0.6%
hbsK75R	0%	3.7%	0.6%	1.9%	1.3%
hbsK80R	0%	6.1%	3.8%	0.9%	0.4%
hbsK86R	0%	1.5%	0.5%	0.6%	0.4%

Average survival at each time point (0, 0.5, 1, 1.5 and 2 hours) of at least three replicates, corresponding to Figure 4B. The standard deviations for each strain and timepoint are displayed. WT: Wildtype.

Supplementary Table S3

	Average				
	0	0.5	1	1.5	2
WT	100%	29.0%	14.0%	7.8%	5.1%
hbsK3Q	100%	9.7%	4.6%	3.5%	2.5%
hbsK3R	100%	3.7%	1.0%	0.9%	0.8%
hbsK41Q	100%	3.0%	2.1%	1.7%	1.0%
hbsK41R	100%	35.8%	28.6%	16.7%	14.4%

	Standard Deviation				
	0	0.5	1	1.5	2
WT	0%	10.6%	6.2%	0.5%	3.1%
hbsK3Q	0%	0.3%	2.3%	2.5%	2.3%
hbsK3R	0%	0.2%	0.1%	0.1%	0.2%
hbsK41Q	0%	0.9%	0.4%	0.6%	0.3%
hbsK41R	0%	4.2%	1.0%	2.9%	7.8%

Average survival at each time point (0, 0.5, 1, 1.5 and 2 hours) of at least three replicates, corresponding to Figure 5. The standard deviations for each strain and timepoint are displayed. WT: Wildtype.

Supplementary Table S4

	Average				
	0	0.5	1	1.5	2
**WT**	100%	95.5%	86.6%	87.4%	80.8%
hbsK3Q	100%	54.0%	37.1%	48.0%	42.1%
hbsK18Q	100%	100.0%	99.9%	91.8%	90.3%
hbsK37Q	100%	90.9%	89.0%	62.3%	49.6%
hbsK41Q	100%	92.0%	99.1%	89.7%	86.3%
hbsK75Q	100%	81.1%	80.0%	38.3%	35.1%
hbsK80Q	100%	10.1%	2.2%	2.2%	1.7%
hbsK86Q	100%	103.2%	83.7%	76.1%	66.2%

	Standard Deviation				
	0	0.5	1	1.5	2
**WT**	0%	11.6%	9.0%	8.8%	7.5%
hbsK3Q	0%	2.8%	19.7%	23.6%	20.8%
hbsK18Q	0%	24.0%	21.4%	5.7%	10.2%
hbsK37Q	0%	12.9%	8.8%	9.9%	13.8%
hbsK41Q	0%	12.6%	1.9%	2.7%	6.2%
hbsK75Q	0%	3.2%	27.1%	16.6%	22.4%
hbsK80Q	0%	1.5%	0.4%	1.0%	1.9%
hbsK86Q	0%	30.2%	24.3%	22.9%	42.6%

Average survival at each time point (0, 0.5, 1, 1.5 and 2 hours) of at least three replicates, corresponding to Figure 6A. The standard deviations for each strain and timepoint are displayed. WT: Wildtype.

Supplementary Table S5

	Average				
	0	0.5	1	1.5	2
**WT**	100%	95.5%	86.6%	87.4%	80.8%
hbsK3Q	100%	54.0%	37.1%	48.0%	42.1%
hbsK18Q	100%	100.0%	99.9%	91.8%	90.3%
hbsK37Q	100%	90.9%	89.0%	62.3%	49.6%
hbsK41Q	100%	92.0%	99.1%	89.7%	86.3%
hbsK75Q	100%	81.1%	80.0%	38.3%	35.1%
hbsK80Q	100%	10.1%	2.2%	2.2%	1.7%
hbsK86Q	100%	103.2%	83.7%	76.1%	66.2%

	Standard Deviation				
	0	0.5	1	1.5	2
**WT**	0%	11.6%	9.0%	8.8%	7.5%
hbsK3R	0%	6.0%	6.8%	6.5%	4.0%
hbsK18R	0%	10.6%	4.6%	2.3%	5.6%
hbsK37R	0%	2.7%	0.9%	1.9%	1.5%
hbsK41R	0%	8.4%	2.3%	1.0%	3.9%
hbsK75R	0%	6.3%	4.1%	2.5%	3.7%
hbsK80R	0%	26.8%	29.9%	12.9%	30.8%
hbsK86R	0%	9.1%	9.7%	15.3%	5.1%

Average survival at each time point (0, 0.5, 1, 1.5 and 2 hours) of at least three replicates, corresponding to Figure 6B. The standard deviations for each strain and timepoint are displayed. WT: Wildtype.
